# Computationally Efficient Nonlinear Model Predictive Control Using the L_1_ Cost-Function

**DOI:** 10.3390/s21175835

**Published:** 2021-08-30

**Authors:** Maciej Ławryńczuk, Robert Nebeluk

**Affiliations:** Institute of Control and Computation Engineering, Faculty of Electronics and Information Technology, Warsaw University of Technology, ul. Nowowiejska 15/19, 00-665 Warsaw, Poland; R.Nebeluk@ia.pw.edu.pl

**Keywords:** process control, model predictive control, L_1_ cost function, optimisation

## Abstract

Model Predictive Control (MPC) algorithms typically use the classical L${}_{2}$ cost function, which minimises squared differences of predicted control errors. Such an approach has good numerical properties, but the L${}_{1}$ norm that measures absolute values of the control errors gives better control quality. If a nonlinear model is used for prediction, the L${}_{1}$ norm leads to a difficult, nonlinear, possibly non-differentiable cost function. A computationally efficient alternative is discussed in this work. The solution used consists of two concepts: (a) a neural approximator is used in place of the non-differentiable absolute value function; (b) an advanced trajectory linearisation is performed on-line. As a result, an easy-to-solve quadratic optimisation task is obtained in place of the nonlinear one. Advantages of the presented solution are discussed for a simulated neutralisation benchmark. It is shown that the obtained trajectories are very similar, practically the same, as those possible in the reference scheme with nonlinear optimisation. Furthermore, the L${}_{1}$ norm even gives better performance than the classical L${}_{2}$ one in terms of the classical control performance indicator that measures squared control errors.

## 1. Introduction

In Model Predictive Control (MPC), a dynamical model of the process is used on-line to repeatedly make predictions of the future values of the controlled variables and to optimise the current and future control policy [1,2]. Due to such a formulation, very good closed-loop control accuracy is obtained and all necessary constraints may be easily imposed on process variables. MPC algorithms are utilised for industrial process control, e.g., chemical reactors [3] and distillation columns [4]. In addition to that, MPC algorithms are also used for fast dynamical systems, e.g., electromagnetic mills [5], electromechanical systems [6], servomotors [7], quadrotors [8], autonomous vehicles [9,10,11], unmanned aerial vehicles [12] and stochastic systems [13]. Four conditions must be fulfilled to obtain good control quality: good measurements, a precise model, an adequate choice of the MPC cost function and a fast MPC computation scheme carried out on-line.

MPC algorithms depend heavily on precise measurements of process variables that are provided by sensors. In some versions of automatic control systems, e.g., those described in [12,14,15], it is stressed that all necessary variables must be measured because otherwise, a significant loss in control performance is unavoidable. If the measurements are not available, the typical approach is to perform on-line estimation using Kalman or Extended Kalman filters [16]. Furthermore, there are other alternative approaches to solve this problem to some extent. They are usually developed for particular applications. For example, when the measurement of lateral vehicle speed is not available, it can be estimated from other measurable parameters [10]. The study presented in [17] shows another approach of dealing with faulty sensors for linear systems by changing the representation of the process model. The work in [18] presents a real vehicle that uses an external camera to detect obstacles and lanes on the road as well as external rear-corner radars to detect objects coming from the rear. An interesting example is presented in [19], where an anemometer is used to measure external disturbances such as wind force and direction. In [20], a depth sensor is installed for sea ship depth measurement. Moreover, the heave speed is obtained by calculating the derivative from depth sensor data. Finally, an example outside the vehicle field where fault-tolerant control is handled using MPC is stiction in control valves [21].

Typically, the minimised cost function used in MPC measures the sum of squared control errors predicted over some time horizon (the MPC-L${}_{2}$ algorithm). Such a formulation is computationally simple and has good numerical properties. The use of the sum of absolute values of the predicted control errors (the MPC-L${}_{1}$ algorithm) is much less popular. However, it may be easily verified that the second approach leads to better control quality [22,23,24,25]; the result is independent of the indicator used [23]. Furthermore, the classical L${}_{2}$ cost function is not always suitable from the point of stability, as pointed out in [26]. It must be also stressed that in regression (model identification), the classical L${}_{2}$ approach yields solutions fragile to outliers, while the L${}_{1}$ norm leads to robustness [27].

It is important to emphasise that the numerical difficulty associated with the MPC-L${}_{1}$ optimisation task depends on the type of dynamical model used for prediction. Provided that a linear model is used, one obtains a linear optimisation task [2,22], which can be solved very efficiently using the classical simplex method. Unfortunately, it is not possible when a nonlinear model is used. In such a case, a nonlinear optimisation problem with constraints must be solved at each sampling instant on-line, which may be computationally too demanding or completely impossible. Examples of nonlinear MPC-L${}_{1}$ algorithms in which the Sequential Quadratic Programming algorithm is used are described in [23,24]. A trust-region sequential quadratic programming method is used in [28].

This work describes computationally efficient approaches to the MPC-L${}_{1}$ algorithms for nonlinear processes. In spite of the fact that a nonlinear model is used for prediction, a computationally simple quadratic optimisation task is solved at each sampling instant on-line, and nonlinear optimisation is not used. The proposed solution is based on two concepts: a neural approximator and advanced on-line linearisation. A practical approach to nonlinear MPC is to perform model or trajectory linearisation on-line. As a result, an easy-to-solve quadratic optimisation problem is obtained. Details of a few such algorithms may be found in [29]. An alternative is to use the fuzzy approach, in which a combination of multiple linear models approximates the nonlinear process, which also results in quadratic optimisation, e.g., [30,31]. Unfortunately, in research carried out so far, only the classical MPC-L${}_{2}$ cost function has been used. The main difficulty is the fact that the cost function in the rudimentary MPC-L${}_{1}$ optimisation task is not differentiable. To make it possible, in the presented approach, the classical MPC-L${}_{1}$ cost function is replaced by its differentiable representation. Such a representation is obtained by means of the neural approximator. Neural networks of the Multi Layer Perceptron (MLP) structure with two layers are used for two reasons. Firstly, they are universal approximators, capable of approximating nonlinear relations with great accuracy. Secondly, the obtained neural approximation is differentiable and poses no numerical problems for the presented computational scheme. The efficiency of the presented approaches is demonstrated for a simulated neutralisation process. In particular, the discussed MPC-L${}_{1}$ algorithms are compared with the classical MPC-L${}_{2}$ ones.

It is worth stressing that different neural structures have been used in MPC for different purposes. First of all, they may be used for prediction:

(a) Neural networks are most often used as black-box models of dynamical processes. Various structures are used: the classical MLP networks [29,32,33], Radial Basis Function (RBF) networks [34,35,36], Long Short-Term Memory (LSTM) [37,38,39] and Gated Recurrent Unit (GRU) [39] structures. Typically, the input–output neural models are used. The state–space neural models [29,40,41] are used when the state–space process description is necessary, although such an approach is significantly less popular.
(b) Neural networks are also used in block-oriented cascade dynamical models, which consist of neural static blocks and linear dynamic blocks connected in series. Hammerstein [42,43,44] and Wiener [45,46,47] models are the most frequently used cascade models.
(c) Quasi-linear neural models [48,49]. In this approach, the dynamical model has the classical linear form, but its parameters depend on the operating point of the process and their values are determined on-line by neural networks.
(d) Neural step response models [50]. In this approach, time-varying coefficients of the model are computed on-line by a neural network.
(e) Neural multi-models [51,52]. In this approach, separate networks calculate the predictions for the consecutive sampling instants over the prediction horizon. As a result, the neural model is not used recurrently, which significantly simplifies training. Additionally, prediction errors are not propagated.
(f) Hybrid neural models [53]. In this approach, neural networks are used to calculate the parameters of the first-principle models.
(g) Neural networks may be used for modelling and MPC of distributed parameter systems [54,55,56].

Additionally, neural networks may be utilised to accelerate and simplify on-line calculations in MPC:

(a) Neural inverse static models are used to try to cancel process nonlinearity. In particular, such a method is frequently used when Wiener cascade models are considered [47,57]. As a result, a quadratic optimisation task is obtained in place of a nonlinear one.
(b) A neural approximator may be used to find the initial solution of the MPC optimisation problem, which speeds up calculations [58,59].
(c) Neural networks are able to approximate the MPC control law [60,61,62]. For training, sufficiently rich data sets are necessary, obtained for different operating points.
(d) Specialised recurrent neural networks may be used to solve the MPC optimisation task on-line [63,64]. As a result, numerical optimisation is not necessary.

The article is organised in the following way. Section 2 recalls the general MPC-L${}_{1}$ and MPC-L${}_{2}$ optimisation tasks. The main part of the article, given in Section 3, details the computationally efficient nonlinear MPC schemes in which the L${}_{1}$ cost function is used. Section 4 thoroughly discusses simulation results for a neutralisation reactor. In particular, MPC-L${}_{1}$ and MPC-L${}_{2}$ algorithms are compared. Finally, Section 5 summarises the whole article.

## 2. Problem Formulation

In this work, MPC of a Single-Input Single-Output processes is considered. The input of the process, i.e., the manipulated variable, is denoted by *u*. The output of the process, i.e., the controlled variable, is denoted by *y*. The vector of decision variables calculated on-line at each sampling instant of MPC is defined as a set of $N_{u}$ increments of the manipulated variable:

(1) $$▵u\left(k\right)=\left[\begin{array}{c} ▵u(k|k)\\ \vdots \\ ▵u(k+N_{u}-1|k) \end{array}\right]$$

where $N_{u}$ is named the control horizon. The decision variables (1) are computed from a constrained MPC optimisation task. The general form of the rudimentary MPC optimisation task considered in this work is:

(2) $$\begin{array}{cc} \; & \min_{▵u(k)}\left\{J(k)\right\}\\ \; & \mathrm{subject}\;\mathrm{to}\\ \; & u^{\min}\leq u\left(k+p|k\right)\leq u^{\max},\;p=0,\ldots ,N_{u}-1\\ \; & ▵u^{\min}\leq ▵u\left(k+p|k\right)\leq ▵u^{\max},\;p=0,\ldots ,N_{u}-1 \end{array}$$

In this work, two types of constraints are considered: the magnitude constraints imposed on the manipulated variable, defined by $u^{\min}$ and $u^{\max}$, and the constraints imposed on the increments of that variable, defined by $▵u^{\min}$ and $▵u^{\max}$. These constraints are considered over the control horizon, $N_{u}$. Having calculated the decision vector (1) from the MPC optimisation problem (2), the first element of the obtained sequence is applied to the process and the whole computational scheme is repeated at the consecutive sampling instant.

The classical minimised cost function used in MPC (the MPC-L${}_{2}$ algorithm) has the form:

(3) $$J_{2}\left(k\right)=\sum_{p=1}^{N}{\left(y^{\mathrm{sp}}\left(k+p|k\right)-\hat{y}\left(k+p|k\right)\right)}^{2}+\lambda \sum_{p=0}^{N_{u}-1}{\left(▵u(k+p|k)\right)}^{2}$$

The first part of the cost function measures the sum of squared control errors predicted over the prediction horizon *N*; the symbols $y^{\mathrm{sp}}\left(k+p|k\right)$ and $\hat{y}\left(k+p|k\right)$ denote the set-point and the predicted value of the controlled variable, respectively, both for the future sampling instant k+p known (it refers to the set-point) or calculated (it refers to the predictions) at the current sampling instant *k*. The predictions, $\hat{y}\left(k+p|k\right)$, are calculated using a dynamical model of the controlled process. The second part of the cost function is used to minimise excessive changes of the manipulated variable; λ is a weighting coefficient. Additionally, it provides good numerical properties.

In the the MPC-L${}_{1}$ algorithm, the sum of absolute values of the predicted control errors over the prediction horizon are taken into account rather than the sum of squared errors. Hence, the minimised cost function is:

(4) $$J_{1}\left(k\right)=\sum_{p=1}^{N}\left|y^{\mathrm{sp}}\left(k+p|k\right)-\hat{y}\left(k+p|k\right)\right|+\lambda \sum_{p=0}^{N_{u}-1}{\left(▵u(k+p|k)\right)}^{2}$$

To penalise significant changes of the manipulated variable and to obtain good numerical properties, the second part of the cost function is the same as in the MPC-L${}_{2}$ formulation [23].

## 3. Computationally Efficient Nonlinear MPC Using the L${}_{1}$ Cost-Function

Let us formulate the MPC-L${}_{1}$ optimisation task. Taking into account the general MPC problem (2) and the MPC-L${}_{1}$ cost function (4), we obtain:

(5) $$\begin{array}{cc} \; & \min_{▵u(k)}\left\{J_{1}\left(k\right)=\sum_{p=1}^{N}\left|y^{\mathrm{sp}}\left(k+p|k\right)-\hat{y}\left(k+p|k\right)\right|+\lambda \sum_{p=0}^{N_{u}-1}{\left(▵u(k+p|k)\right)}^{2}\right\}\\ \; & \mathrm{subject}\;\mathrm{to}\\ \; & u^{\min}\leq u\left(k+p|k\right)\leq u^{\max},\;p=0,\ldots ,N_{u}-1\\ \; & ▵u^{\min}\leq ▵u\left(k+p|k\right)\leq ▵u^{\max},\;p=0,\ldots ,N_{u}-1 \end{array}$$

The use of a nonlinear model for prediction leads to two computational difficulties. Firstly, predictions $\hat{y}\left(k+p|k\right)$ are nonlinear functions of the calculated decision vector (1), which means that the cost function is nonlinear. As a result, we obtain a nonlinear optimisation task that must be repeated at each sampling instant. Secondly, the absolute value function is not differentiable, which means that the classical gradient-based optimisation method cannot be used. In spite of the second of the mentioned computational difficulties, in the literature, is it possible to find applications of gradient-based nonlinear optimisation methods to solve the MPC-L${}_{1}$ optimisation task (5) [23,24,28]. The objective of this work is to derive a much more computationally simple approach to the MPC-L${}_{1}$ problem in which nonlinear optimisation is not used. To achieve this result, two concepts are used:

(a) The first part of the non-differentiable cost function (4) is replaced by its differentiable representation. For this purpose, a neural network approximation of the absolute value function is used.
(b) The $J_{1}\left(k\right)$ cost function with a neural approximator is differentiable but nonlinear in terms of the computed control moves (1). To simplify the calculation scheme, an advanced trajectory linearisation method is used. As a result, a simple-to-solve quadratic optimisation task is obtained in place of the nonlinear one. Quadratic optimisation problems, for λ>0, have only one minimum, which is the global one.

### 3.1. Neural Approximation of the MPC-L${}_{1}$ Cost-Function

Only the first part of the cost function $J_{1}\left(k\right)$ defined by Equation (4) is not differentiable. Hence, a differentiable approximator of the first part is only necessary. At first, let us define the predicted control error for the future sampling instant k+p computed at the current instant *k*:

(6) $$e\left(k+p|k\right)=y^{\mathrm{sp}}\left(k+p|k\right)-\hat{y}\left(k+p|k\right)$$

where p=1,…,N. Hence, the cost function $J_{1}\left(k\right)$ may be rewritten compactly:

(7) $$J_{1}\left(k\right)=\sum_{p=1}^{N}\left|e(k+p|k)\right|+\lambda \sum_{p=0}^{N_{u}-1}{\left(▵u(k+p|k)\right)}^{2}$$

In order to use quadratic optimisation in MPC, it is postulated to use the general form of the differentiable approximation of the cost function (7):

(8) $$J_{1}\left(k\right)=\sum_{p=1}^{N}{\left(\alpha \left(e\left(k+p|k\right)\right)\right)}^{2}+\lambda \sum_{p=0}^{N_{u}-1}{\left(▵u(k+p|k)\right)}^{2}$$

where α(e(k+p|k)) describes some nonlinear function of the predicted control error. Comparing the cost functions (7) and (8), it is clear that the following approximation of the absolute value function is required:

(9) $${\left(\alpha \left(e\left(k+p|k\right)\right)\right)}^{2}=\left|e\left(k+p|k\right)\right|$$

for all p=1,…,N. In order to obtain a differentiable approximation of the absolute value function, the neural network of a Multi Layer Perceptron (MLP) type is used [65]. The network has two layers, the first of which (the hidden one) is nonlinear, and a linear output. It is defined by the following equation:

(10) $$\alpha \left(e\left(k+p|k\right)\right)=w_{0}^{2}+\sum_{i=1}^{K}w_{i}^{2}\varphi \left[w_{i,0}^{1}+w_{i,1}^{1}\left(e\left(k+p|k\right)\right)\right]$$

where *K* denotes the number of hidden neurons and φ is a nonlinear activation function. The weights of the first layer of the network are denoted by $w_{i,0}^{1}$ and $w_{i,1}^{1}$ for i=1,…,K. The weights of the second layer are denoted by $w_{0}^{2}$ and $w_{i}^{2}$ for i=1,…,K. Provided that a differentiable activation function φ is used, the function α(e(k+p|k)) is also differentiable. It is of course true for the tanh function, which is used in this work.

### 3.2. Advanced Trajectory Linearisation of the MPC-L${}_{1}$ Cost-Function

Thanks to the use of neural approximation (10) of the absolute value function, the cost function $J_{1}\left(k\right)$ defined by Equation (8) is differentiable, but still nonlinear. Additionally, the model of the controlled process is nonlinear. As a result, the cost function $J_{1}\left(k\right)$ is nonlinear in terms of the calculated future control increments (1). To simplify the problem, we use the advanced on-line trajectory linearisation approach. Linearisation is not carried out in a simple way, for the current or past operating point of the process, but for some assumed future trajectory of the manipulated variable, defined over the control horizon:

(11) $$u^{\mathrm{traj}}\left(k\right)=\left[\begin{array}{c} u^{\mathrm{traj}}\left(k|k\right)\\ \vdots \\ u^{\mathrm{traj}}\left(k+N_{u}-1|k\right) \end{array}\right]$$

Using the Taylor series expansion formula, the linear approximation of the multivariable function α(e(k+p|k)) that has $N_{u}$ arguments, defined by the vector $u^{\mathrm{traj}}\left(k\right)$, is:

(12) $$\begin{array}{cc} \alpha (e(k+p|k)) & =\alpha (e^{\mathrm{traj}}\left(k+p|k\right))\\ \; & \;+\sum_{r=0}^{N_{u}-1}\frac{\partial \alpha (e^{\mathrm{traj}}\left(k+p|k\right))}{\partial u^{\mathrm{traj}}\left(k+r|k\right)}\left(u\left(k+r|k\right)-u^{\mathrm{traj}}\left(k+r|k\right)\right) \end{array}$$

for p=1,…,N. In order to find the partial derivatives in the right side of Equation (12), the neural approximator defined by Equation (10) is differentiated with respect to the assumed future trajectory of the manipulated variable (11), which gives:

(13) $$\frac{\partial \alpha (e^{\mathrm{traj}}\left(k+p|k\right))}{\partial u^{\mathrm{traj}}\left(k+r|k\right)}=\sum_{i=1}^{K}w_{i}^{2}\frac{d\varphi (z_{i}^{\mathrm{traj}}\left(k+p|k\right))}{dz_{i}^{\mathrm{traj}}\left(k+p|k\right)}\frac{\partial z_{i}^{\mathrm{traj}}\left(k+p|k\right)}{\partial u^{\mathrm{traj}}\left(k+r|k\right)}$$

for all p=1,…,N, $r=0,\ldots ,N_{u}-1$. The inputs of the hidden nodes of the neural network are:

(14) $$z_{i}^{\mathrm{traj}}\left(k+p|k\right)=w_{i,0}^{1}+w_{i,1}^{1}\left(y^{\mathrm{sp}}\left(k+p|k\right)-{\hat{y}}^{\mathrm{traj}}\left(k+p|k\right)\right)$$

where i=1,…,K, p=1,…,N. If the tanh function is used in the hidden layer of the neural network:

(15) $$\frac{d\varphi (z_{i}^{\mathrm{traj}}\left(k+p|k\right))}{dz_{i}^{\mathrm{traj}}\left(k+p|k\right)}=1-{\left(\varphi \left(z_{i}^{\mathrm{traj}}\left(k+p|k\right)\right)\right)}^{2}$$

Finally, from Equations (13)–(15), we obtain:

(16) $$\frac{\partial \alpha (e^{\mathrm{traj}}\left(k+p|k\right))}{\partial u^{\mathrm{traj}}\left(k+r|k\right)}=-\sum_{i=1}^{K}w_{i,1}^{1}w_{i}^{2}\left(1-{\left(\varphi \left(z_{i}^{\mathrm{traj}}\left(k+p|k\right)\right)\right)}^{2}\right)\frac{\partial {\hat{y}}^{\mathrm{traj}}\left(k+p|k\right)}{\partial u^{\mathrm{traj}}\left(k+r|k\right)}$$

for all p=1,…,N, $r=0,\ldots ,N_{u}-1$. The partial derivatives in the right side of Equation (16) are derived for a particular model structure used for prediction. Differentiability of the model is required.

Let us stress that in Equation (12), independent linear approximations are obtained for the consecutive sampling instants over the prediction horizon, i.e., for p=1,…,N. In MPC, we need approximations of the absolute value of the predicted error over the whole prediction horizon. In order to simplify derivations, a compact vector matrix notation is used. The predicted trajectory of the function α, i.e., the vector form of Equation (12), is the following:

(17) $$\alpha \left(k\right)=\alpha \left(e^{\mathrm{traj}}\left(k\right)\right)+\frac{d\alpha (e^{\mathrm{traj}}\left(k\right))}{du^{\mathrm{traj}}\left(k\right)}\left(u\left(k\right)-u^{\mathrm{traj}}\left(k\right)\right)$$

where vectors of length *N* have the forms:

(18) $$\alpha \left(k\right)=\left[\begin{array}{c} \alpha (e(k+1|k))\\ \vdots \\ \alpha (e(k+N|k)) \end{array}\right]$$

and

(19) $$\alpha \left(e^{\mathrm{traj}}\left(k\right)\right)=\left[\begin{array}{c} \alpha (e^{\mathrm{traj}}\left(k+1|k\right))\\ \vdots \\ \alpha (e^{\mathrm{traj}}\left(k+N|k\right)) \end{array}\right]$$

the vector of length $N_{u}$, corresponding to the vector of increments (1), is:

(20) $$u\left(k\right)=\left[\begin{array}{c} u(k|k)\\ \vdots \\ u(k+N_{u}-1|k) \end{array}\right]$$

and the $N\times N_{u}$ matrix of partial derivatives has the structure:

(21) $$\frac{d\alpha (e^{\mathrm{traj}}\left(k\right))}{du^{\mathrm{traj}}\left(k\right)}=\left[\begin{array}{ccc} \frac{\partial \alpha (e^{\mathrm{traj}}\left(k+1|k\right)}{\partial u^{\mathrm{traj}}\left(k|k\right)} & \cdots & \frac{\partial \alpha (e^{\mathrm{traj}}\left(k+1|k\right)}{\partial u^{\mathrm{traj}}\left(k+N_{u}-1|k\right)}\\ \vdots & \ddots & \vdots \\ \frac{\partial \alpha (e^{\mathrm{traj}}\left(k+N|k\right)}{\partial u^{\mathrm{traj}}\left(k|k\right)} & \cdots & \frac{\partial \alpha (e^{\mathrm{traj}}\left(k+N|k\right)}{\partial u^{\mathrm{traj}}\left(k+N_{u}-1|k\right)} \end{array}\right]$$

The matrix (21), where its entries are defined by Equation (16), is calculated for the specific neural approximator and the nonlinear model used. Because in MPC we calculate not the future values of the manipulated variables (Equation (20)) but the corresponding increments (1), we have to express the vector equation of the linearised trajectory (Equation (17)) in terms of the vector of control increments ▵u(k). We obtain:

(22) $$\alpha \left(k\right)=\frac{d\alpha (e^{\mathrm{traj}}\left(k\right))}{du^{\mathrm{traj}}\left(k\right)}J▵u\left(k\right)+\alpha \left(e^{\mathrm{traj}}\left(k\right)\right)+\frac{d\alpha (e^{\mathrm{traj}}\left(k\right))}{du^{\mathrm{traj}}\left(k\right)}\left(u\left(k-1\right)-u^{\mathrm{traj}}\left(k\right)\right)$$

where the auxiliary matrix of dimensionality $N_{u}\times N_{u}$ is:

(23) $$J=\left[\begin{array}{ccccc} 1 & 0 & 0 & \ldots & 0\\ 1 & 1 & 0 & \ldots & 0\\ \vdots & \vdots & \vdots & \ddots & \vdots \\ 1 & 1\; & 1 & \ldots & 1 \end{array}\right]$$

and the vector of length $N_{u}$ has the structure:

(24) $$u\left(k-1\right)=\left[\begin{array}{c} u(k-1)\\ \vdots \\ u(k-1) \end{array}\right]$$

### 3.3. Formulation of the Computationally Simple MPC-L${}_{1}$ Quadratic Optimisation Task

In order to obtain a quadratic optimisation MPC-L${}_{1}$ optimisation problem, we take into account the general nonlinear MPC-L${}_{1}$ optimisation task defined by Equation (5) in which the first part of the minimised cost function is approximated by Equation (22). As a result, we obtain:

(25) $$\begin{array}{cc} \; & \min_{▵u(k)}\{J_{1}\left(k\right)=∥\frac{d\alpha (e^{\mathrm{traj}}\left(k\right))}{du^{\mathrm{traj}}\left(k\right)}J▵u\left(k\right)+\alpha \left(e^{\mathrm{traj}}\left(k\right)\right)\\ \; & \;\;\;\;+\frac{d\alpha (e^{\mathrm{traj}}\left(k\right))}{du^{\mathrm{traj}}\left(k\right)}\left(u\left(k-1\right)-u^{\mathrm{traj}}\left(k\right)\right){∥}^{2}+{∥▵u(k)∥}_{\Lambda }^{2}\}\\ \; & \mathrm{subject}\;\mathrm{to}\\ \; & u^{\min}\leq J▵u\left(k\right)+u\left(k-1\right)\leq u^{\max}\\ \; & ▵u^{\min}\leq ▵u\left(k\right)\leq ▵u^{\max} \end{array}$$

The matrix Λ=diag(λ,…,λ) is of dimensionality $N_{u}\times N_{u}$. Thanks to linearisation, the minimised cost function is quadratic in terms of the decision vector, ▵u(k), and all the constraints are linear with respect to the vector ▵u(k). The auxiliary vectors of length $N_{u}$ are:

(26) $$u^{\min}=\left[\begin{array}{c} u^{\min}\\ \vdots \\ u^{\min} \end{array}\right],\;u^{\max}=\left[\begin{array}{c} u^{\max}\\ \vdots \\ u^{\max} \end{array}\right],\;▵u^{\min}=\left[\begin{array}{c} ▵u^{\min}\\ \vdots \\ ▵u^{\min} \end{array}\right],\;▵u^{\max}=\left[\begin{array}{c} ▵u^{\max}\\ \vdots \\ ▵u^{\max} \end{array}\right]$$

The obtained optimisation problem (25) can now be transformed into the standard form, typical of quadratic optimisation tasks:

(27) $$\begin{array}{cc} \; & \min_{x(k)}\left\{0.5x^{T}\left(k\right)H_{\mathrm{QP}}\left(k\right)x\left(k\right)+f_{\mathrm{QP}}^{T}\left(k\right)x\left(k\right)\right\}\\ \; & \mathrm{subject}\;\mathrm{to}\\ \; & A(k)x(k)\leq b(k)\\ \; & \mathrm{LB}\leq x(k)\leq \mathrm{UB} \end{array}$$

where the inequality constraints are defined by the matrix:

(28) $$\begin{array}{cc} A(k) & =\left[\begin{array}{c} -J\\ J \end{array}\right] \end{array}$$

and the vector:

(29) $$\begin{array}{cc} b(k) & =\left[\begin{array}{c} -u^{\min}+u\left(k-1\right)\\ u^{\max}-u\left(k-1\right) \end{array}\right] \end{array}$$

while the box constraints imposed on the decision vector are:

(30) $$\mathrm{LB}=▵u^{\min},\;\mathrm{UB}=▵u^{\max}$$

By differentiating the cost function $J_{1}\left(k\right)$ used in the quadratic optimisation problem (25) with respect to the decision variables, ▵u(k), we obtain:

(31) $$\begin{array}{cc} \frac{dJ(k)}{d▵u(k)} & =2\left(J^{T}{\left(\frac{d\alpha (e^{\mathrm{traj}}\left(k\right))}{du^{\mathrm{traj}}\left(k\right)}\right)}^{T}\frac{d\alpha (e^{\mathrm{traj}}\left(k\right))}{du^{\mathrm{traj}}\left(k\right)}J+\Lambda \right)▵u\left(k\right)\\ \; & \;+2J^{T}{\left(\frac{d\alpha (e^{\mathrm{traj}}\left(k\right))}{du^{\mathrm{traj}}\left(k\right)}\right)}^{T}(\alpha \left(e^{\mathrm{traj}}\left(k\right)\right)\\ \; & \;\;\;\;\;\;+\frac{d\alpha (e^{\mathrm{traj}}\left(k\right))}{du^{\mathrm{traj}}\left(k\right)}\left(u\left(k-1\right)-u^{\mathrm{traj}}\left(k\right)\right)) \end{array}$$

Hence, the Hessian matrix necessary in the classical form of the quadratic optimisation task (27) is:

(32) $$H_{\mathrm{QP}}\left(k\right)=2J^{T}{\left(\frac{d\alpha (e^{\mathrm{traj}}\left(k\right))}{du^{\mathrm{traj}}\left(k\right)}\right)}^{T}\frac{d\alpha (e^{\mathrm{traj}}\left(k\right))}{du^{\mathrm{traj}}\left(k\right)}J+2\Lambda$$

while the vector $f_{\mathrm{QP}}$ is:

(33) $$f_{\mathrm{QP}}\left(k\right)=2J^{T}{\left(\frac{d\alpha (e^{\mathrm{traj}}\left(k\right))}{du^{\mathrm{traj}}\left(k\right)}\right)}^{T}\left(\alpha \left(e^{\mathrm{traj}}\left(k\right)\right)+\frac{d\alpha (e^{\mathrm{traj}}\left(k\right))}{du^{\mathrm{traj}}\left(k\right)}\left(u\left(k-1\right)-u^{\mathrm{traj}}\left(k\right)\right)\right)$$

Calculation of the matrix of derivatives, as well as the trajectory, are explained for the specific form of the model used in simulations presented in Section 4.

In general, two versions of the presented algorithm are possible. Firstly, in the MPC algorithm with nonlinear prediction and linearisation along the trajectory (MPC-NPLT-L${}_{1}$), trajectory linearisation may be executed once at each sampling instant for the assumed trajectory $u^{\mathrm{traj}}\left(k\right)$. It means that only one quadratic optimisation problem is solved at every sampling instant. Alternatively, in the MPC algorithm with nonlinear prediction and linearisation along the predicted trajectory (MPC-NPLPT-L${}_{1}$), a few internal iterations are possible at each sampling instant. The first internal iteration is the same as in the MPC-NPLT-L${}_{1}$ scheme. In the consecutive ones, the optimal solution obtained in the previous internal iteration is used for linearisation.

## 4. Simulations

### 4.1. The Neutralisation Reactor

In this work, the neutralisation reactor is used as a benchmark to evaluate and compare all considered MPC methods. The process has one manipulated variable, which is a base (NaOH) stream $q_{1}$ (mL/s) and one controlled variable, which is the value of pH of the product. The detailed fundamental model of the process is given in [66]. The process is nonlinear, since its static and dynamic properties depend on the operating point. Hence, it is frequently used as a good benchmark to evaluate model identification algorithms and advanced nonlinear control methods.

### 4.2. Neutralisation Reactor Modelling for MPC

In this work, a Wiener model [67] of the neutralisation reactor is used in MPC for prediction. Such a block-orientated model is composed of two parts: a linear dynamic block followed by a nonlinear static one. The first part of the Wiener model is described by second-order dynamics:

(34) $$v\left(k\right)=b_{1}u\left(k-1\right)+b_{2}u\left(k-2\right)-a_{1}v\left(k-1\right)-a_{2}v\left(k-2\right)$$

The auxiliary variable between two model blocks is denoted by *v*. The second part of the model is represented by a differentiable function:

(35) y(k)=g(v(k))

Combining Equations (34) and (35), we obtain the output of the Wiener model for the sampling instant *k*:

(36) $$y\left(k\right)=g\left(v\left(k\right)\right)=g\left(b_{1}u\left(k-1\right)+b_{2}u\left(k-2\right)-a_{1}v\left(k-1\right)-a_{2}v\left(k-2\right)\right)$$

The relations between the process input and output variables, i.e., $q_{1}$ and pH, respectively, and the input and output of the model, i.e., *u* and *y*, respectively, are given by the following relations:

(37) $$u=q_{1}-q_{1,0},\;y=\mathrm{pH}-{\mathrm{pH}}_{0}$$

where in the nominal operating point, $q_{1,0}=15.5$ and ${\mathrm{pH}}_{0}=7$. In this study, a sigmoid-like neural network is used to represent the nonlinear static part of the model. Details of model training, validation and selection are given in [68]. An alternative, a Support Vector Machine (SVM) Wiener model, is presented in [69]. The sampling time of the Wiener model is 10 s.

### 4.3. Calculation of the Predicted Trajectories for the Wiener Model of the Neutralisation Reactor

The trajectory $\alpha (e^{\mathrm{traj}}\left(k\right))$, defined by Equation (19), is calculated using the neural approximator from Equation (10). The predicted control errors, e(k+p|k), are computed from Equation (6). The predicted values of the controlled variable, ${\hat{y}}^{\mathrm{traj}}\left(k+p|k\right)$, are calculated from the Wiener model in the following way. From the linear block of the model (Equation (34)), we have:

(38) $$\begin{array}{cc} v^{\mathrm{traj}}\left(k+1|k\right) & =b_{1}u^{\mathrm{traj}}\left(k|k\right)+b_{2}u\left(k-1\right)-a_{1}v\left(k\right)-a_{2}v\left(k-1\right) \end{array}$$

(39) $$\begin{array}{cc} v^{\mathrm{traj}}\left(k+2|k\right) & =b_{1}u^{\mathrm{traj}}\left(k+1|k\right)+b_{2}u^{\mathrm{traj}}\left(k|k\right)-a_{1}v^{\mathrm{traj}}\left(k+1|k\right)-a_{2}v\left(k\right)\\ \; & \vdots \\ v^{\mathrm{traj}}\left(k+p|k\right) & =b_{1}u^{\mathrm{traj}}\left(k-1+p|k\right)+b_{2}u^{\mathrm{traj}}\left(k-2+p|k\right) \end{array}$$

(40) $$\begin{array}{cc} \; & \;-a_{1}v^{\mathrm{traj}}\left(k-1+1|k\right)-a_{2}v^{\mathrm{traj}}\left(k-2+p|k\right),\;p=3,\ldots ,N \end{array}$$

From the nonlinear block of the model (Equation (36)), the output predictions are:

(41) $$\begin{array}{cc} {\hat{y}}^{\mathrm{traj}}\left(k+p|k\right) & =g(v^{\mathrm{traj}}\left(k+p|k\right))+d\left(k\right) \end{array}$$

for any sampling time instant k+p|k, where p=1,…,N. The disturbance estimate is calculated using Equation (36), which yields:

(42) $$d\left(k\right)=y\left(k\right)-g(b_{1}u\left(k-1\right)+b_{2}u\left(k-2\right)-a_{1}v\left(k-1\right)-a_{2}v\left(k-2\right))$$

### 4.4. Calculation of the Matrices of Derivatives for the Wiener Model of the Neutralisation Reactor

The elements of the matrix $\frac{d\alpha (e^{\mathrm{traj}}\left(k\right))}{du^{\mathrm{traj}}\left(k\right)}$ defined by Equation (21) can be found using Equation (16). The derivatives $\frac{\partial {\hat{y}}^{\mathrm{traj}}\left(k+p|k\right)}{\partial u^{\mathrm{traj}}\left(k+r|k\right)}$ for all p=1,…,N and $r=0,\ldots ,N_{u}-1$ are calculated for the Wiener model used in the following way. By differentiating Equation (41), the following formula is obtained:

(43) $$\frac{\partial {\hat{y}}^{\mathrm{traj}}\left(k+p|k\right)}{\partial u^{\mathrm{traj}}\left(k+r|k\right)}=\frac{dg(v^{\mathrm{traj}}\left(k+p|k\right))}{dv^{\mathrm{traj}}\left(k+p|k\right)}\frac{\partial v^{\mathrm{traj}}\left(k+p|k\right)}{\partial u^{\mathrm{traj}}\left(k+r|k\right)}$$

The first derivative in Equation (43) depends on the type of nonlinear static function. The second is calculated recursively for k+1|k,…,k+N|k. For the first sampling time instant, k+1, calculated at the current time instant, *k*; by differentiating the formula (38), the following formula is acquired:

(44) $$\begin{array}{c} \frac{\partial v^{\mathrm{traj}}\left(k+1|k\right)}{\partial u^{\mathrm{traj}}\left(k+r|k\right)}=\left\{\begin{array}{cc} b_{1} & \mathrm{for}r=0\\ 0 & \mathrm{for}r>0 \end{array}\right. \end{array}$$

Prediction $\hat{y}\left(k+1|k\right)$ does not depend on control signals u(k+1|k),u(k+2|k),…. With that in mind, the following formula is obtained:

(45) $$\frac{\partial {\hat{y}}^{\mathrm{traj}}\left(k+1|k\right)}{\partial u^{\mathrm{traj}}\left(k+r|k\right)}=0\;\mathrm{for}\;\mathrm{all}\;r>0$$

For the next time instant, k+2, by differentiating Equation (39), the following is acquired:

(46) $$\begin{array}{cc} \frac{\partial v^{\mathrm{traj}}\left(k+2|k\right)}{\partial u^{\mathrm{traj}}\left(k+r|k\right)} & =b_{1}\frac{\partial u^{\mathrm{traj}}\left(k+1|k\right)}{\partial u^{\mathrm{traj}}\left(k+r|k\right)}+b_{2}\frac{\partial u^{\mathrm{traj}}\left(k|k\right)}{\partial u^{\mathrm{traj}}\left(k+r|k\right)}-a_{1}\frac{\partial v^{\mathrm{traj}}\left(k+1|k\right)}{\partial u(k+r|k)} \end{array}$$

where the derivatives $\frac{\partial u^{\mathrm{traj}}\left(k+p|k\right)}{\partial u^{\mathrm{traj}}\left(k+r|k\right)}$ can only take the values 0 or 1:

(47) $$\frac{\partial u^{\mathrm{traj}}\left(k+p|k\right)}{\partial u^{\mathrm{traj}}\left(k+r|k\right)}=\left\{\begin{array}{cc} 1 & \mathrm{for}p=r\mathrm{or}(p>r\mathrm{and}r=N_{u}-1)\\ 0 & \mathrm{otherwise} \end{array}\right.$$

For the time instant k+p|k, where p=3,…,N, by differentiating Equation (40), we obtain:

(48) $$\begin{array}{cc} \frac{\partial v^{\mathrm{traj}}\left(k+p|k\right)}{\partial u^{\mathrm{traj}}\left(k+r|k\right)} & =b_{1}\frac{\partial u^{\mathrm{traj}}\left(k-1+p|k\right)}{\partial u^{\mathrm{traj}}\left(k+r|k\right)}+b_{2}\frac{\partial u^{\mathrm{traj}}\left(k-2+p|k\right)}{\partial u^{\mathrm{traj}}\left(k+r|k\right)}\\ \; & \;-a_{1}\frac{\partial v^{\mathrm{traj}}\left(k-1+p|k\right)}{\partial u^{\mathrm{traj}}\left(k+r|k\right)}-a_{2}\frac{\partial v^{\mathrm{traj}}\left(k-2+p|k\right)}{\partial u^{\mathrm{traj}}\left(k+r|k\right)} \end{array}$$

Analogously, using Equation (45), a general regularity can be observed:

(49) $$\frac{\partial {\hat{y}}^{\mathrm{traj}}\left(k+p|k\right)}{\partial u^{\mathrm{traj}}\left(k+r|k\right)}=0\;\mathrm{for}\;r\geq p$$

### 4.5. Organisation of Calculations

1. For the Wiener model, the disturbance estimate is calculated from Equation (42).
2. The trajectory of the manipulated variable, $u^{\mathrm{traj}}\left(k\right)$, that defines the linearisation point (Equation (11)), is formed. Three possible choices are discussed in the next section.
3. For the Wiener model, the derivatives $\frac{\partial {\hat{y}}^{\mathrm{traj}}\left(k+p|k\right)}{\partial u^{\mathrm{traj}}\left(k+r|k\right)}$, for all p=1,…,N and r=0,…,$N_{u}-1$, are calculated using Equations (43)–(49).
4. The matrix $\frac{d\alpha (e^{\mathrm{traj}}\left(k\right))}{du^{\mathrm{traj}}\left(k\right)}$ defined by Equation (21) is calculated using Equation (16).
5. The quadratic optimisation task (25) is solved.
6. In the case of the MPC-NPLT-L${}_{1}$ algorithm, the first element of the obtained decision vector, $▵u^{\mathrm{opt}}\left(k\right)$, is applied to the process, i.e., $u\left(k\right)=▵u^{\mathrm{opt}}\left(k|k\right)+u\left(k-1\right)$.
7. In the case of the MPC-NPLPT-L${}_{1}$ algorithm, steps 2–5 are repeated a few times (in this work, maximally five times). The trajectory used for linearisation is defined as $u^{\mathrm{traj}}\left(k\right)=J▵u^{\mathrm{opt}}\left(k\right)+u\left(k-1\right)$, where the matrix J and the vector u(k−1) are defined by Equations (23) and (24), respectively, and $▵u^{\mathrm{opt}}\left(k\right)$ denotes the optimal solution calculated in the previous internal iteration (for the current sampling instant *k*). When the internal iterations are terminated, the first element of the decision vector computed in the last internal iteration is applied to the process.

### 4.6. Comparison of MPC-L${}_{1}$ and MPC-L${}_{2}$ Algorithms for the Neutralisation Reactor

**Figure 1:**
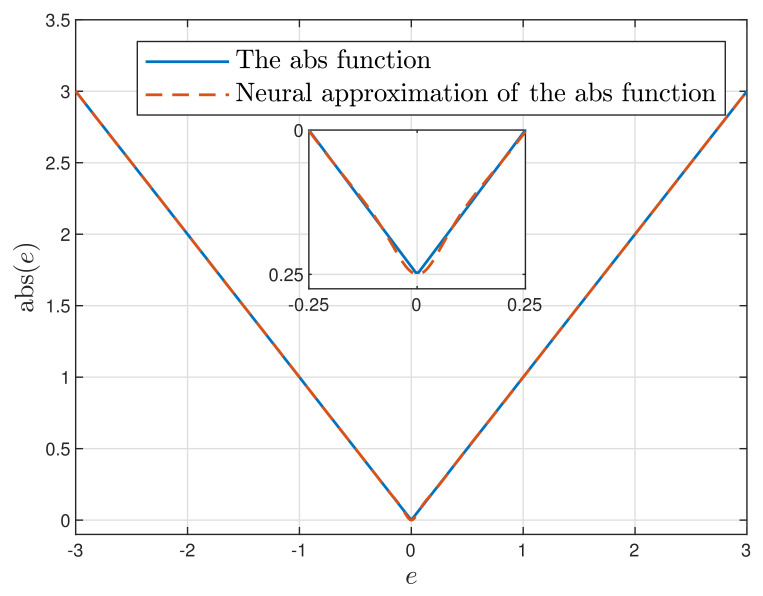
The absolute value function vs. its neural approximation.

At first, let us verify the usefulness of the MLP neural network to serve as an approximator of the absolute value function of the predicted control error (Equation (10)). The neural network with K=10 hidden nodes of the tanh type is used. Figure 1 compares the non-differentiable absolute value (abs) function and its differentiable neural approximation. The range of the control error, *e*, is adequate for further use of the neural approximator in MPC for the considered neutralisation reactor. For the chosen neural network structure and the number of hidden nodes, the obtained approximation accuracy is very good.

In the following part of the article, the following MPC algorithms are considered:

- MPC-NO-L${}_{1}$: the MPC algorithm with nonlinear optimisation with the L${}_{1}$ norm used in the first part of the minimised cost function defined by Equation (4). The resulting nonlinear optimisation task is given by Equation (5). Two versions of the MPC-NO-L${}_{1}$ are considered: the non-differentiable absolute value function or its differentiable neural approximation may be used.
- MPC-NPLT1-L${}_{1}$: the discussed MPC algorithm with nonlinear prediction and linearisation along the trajectory. The neural network is used to approximate the non-differentiable absolute value function. Moreover, a linear approximation of the nonlinear trajectory of the predicted control errors over the prediction horizon is used in the cost function. The resulting quadratic optimisation task is given by Equation (25). The trajectory used for linearisation, i.e., $u^{\mathrm{traj}}\left(k\right)$ (Equation (11)) is constant; all its elements are equal to the value of the manipulated variable calculated at the previous sampling instant, i.e., u(k−1), and applied to the process.
- MPC-NPLT2-L${}_{1}$: the trajectory used for linearisation is defined by the last $N_{u}-1$ elements of the optimal solution ▵u(k) computed at the previous sampling instant. Only the first element of this sequence is actually used for control.
- MPC-NPLT3-L${}_{1}$: the trajectory used for linearisation is constant, all its elements are equal to the value of the process input corresponding to the current output set-point. For this purpose, the inverse static model of the process is used: $u^{\mathrm{sp}}\left(k\right)=\tilde{g}\left(y^{\mathrm{sp}}\left(k\right)\right)$. In this work, a neural network of the MLP type with two layers serves as the inverse model (the first nonlinear layer contains 10 hidden nodes of the tanh type).
- MPC-NPLPT-L${}_{1}$: the discussed MPC algorithm with nonlinear prediction and linearisation along the predicted trajectory. In this case, trajectory linearisation and quadratic optimisation are repeated maximally five times at each sampling instant. The trajectory used for linearisation is taken from the previous internal iteration of the algorithm. In the first internal iteration, for linearisation, the trajectory obtained from the inverse static model for the current set-point is used, exactly as it is done in the MPC-NPLT3-L${}_{1}$ scheme.

Similarly, the following MPC algorithms with the L${}_{2}$ cost function (3) are considered:

- MPC-NO-L${}_{2}$: the MPC algorithm with nonlinear optimisation with the L${}_{2}$ norm used in two parts of the minimised cost function. The resulting nonlinear optimisation task is given by Equation (2).
- MPC-NPLT1-L${}_{2}$, MPC-NPLT2-L${}_{2}$ and MPC-NPLT3-L${}_{2}$: the MPC algorithm with nonlinear prediction and linearisation along the trajectory [29]. Trajectory linearisation and quadratic optimisation are performed once at each sampling instant.
- MPC-NPLPT-L${}_{2}$: the MPC algorithm with Nonlinear Prediction and Linearisation along the Predicted Trajectory [29]. Trajectory linearisation and quadratic optimisation are repeated maximally five times at each sampling instant.

In all simulations presented next, the same parameters of MPC are used: N=10, $N_{u}=3$, λ=1. The constraints are imposed on the magnitude of the manipulated variable: $q_{1}^{\min}=0$, $q_{1}^{\max}=30$. All simulations are performed in MATLAB. The fmincon and quadprog functions are used for nonlinear and quadratic optimisation, respectively; default parameters are used in both cases.

**Figure 2:**
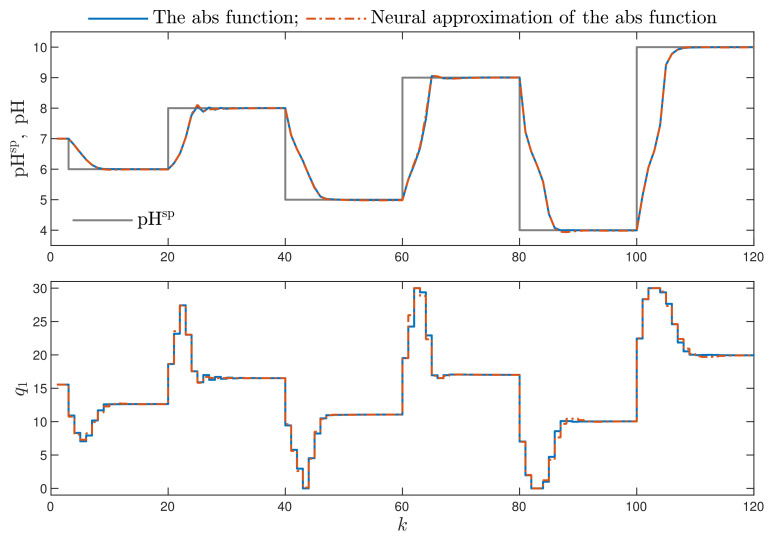

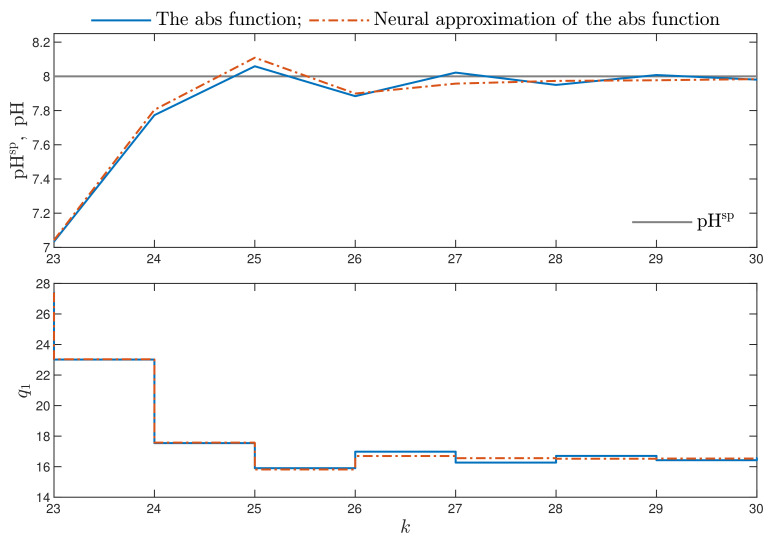
Simulation results: the MPC-NO-L${}_{1}$ algorithm using the non-differentiable absolute value function vs. the MPC-NO-L${}_{1}$ algorithm using the neural approximation of the absolute value function; two top panels show the results for the whole simulation horizon, two bottom panels show an enlarged fragment for the sampling instants 23≤k≤30.

Firstly, let us evaluate the efficiency of two versions of the MPC-NO-L${}_{1}$ control scheme. In both of them, nonlinear optimisation is used at each sampling instant on-line, but the objective of the comparison is to only check the efficiency of the neural approximator of the absolute value function. In the first case, the non-differentiable absolute value function is used in the first part of the minimised cost function, while in the second case, a neural approximator is considered. Figure 2 depicts the obtained results for a few changes of the set-point. In general, the non-differentiable absolute value function results in some numerical problems for the optimisation routine. The influence of such problems on the resulting trajectories of the process is best visible for the second step of the set-point. For better comparison, two bottom panels of Figure 2 show an enlarged fragment of the obtained results. For the non-differentiable absolute value function, the sign of the real control error changes a few times, while the neural approximator smooths the trajectory of the process output.

**Figure 3:**
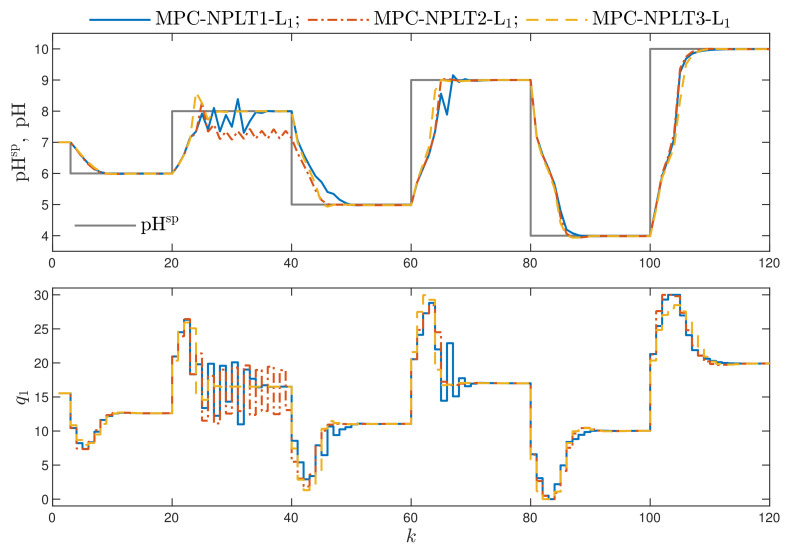
Simulation results: the MPC-NPLT1-L${}_{1}$, MPC-NPLT2-L${}_{1}$ and MPC-NPLT3-L${}_{1}$ algorithms.

Figure 3 compares the simulation results of three computationally efficient MPC algorithms with one repetition of trajectory linearisation and quadratic optimisation at each sampling instant, but the trajectory used for linearisation, i.e., $u^{\mathrm{traj}}\left(k\right)$, is chosen in different ways. In all algorithms, the L${}_{1}$ norm cost function is used. The first two initialisation methods, used in the MPC-NPLT1-L${}_{1}$ and MPC-NPLT2-L${}_{1}$ algorithms, lead to some problems for the second set-point step. The explanation is the following: when the set-point changes abruptly, the linear approximation of the predicted trajectory performed using the past operating point (MPC-NPLT1-L${}_{1}$) or the part of the optimal trajectory for the past operating point (MPC-NPLT2-L${}_{1}$) differs significantly from the true nonlinear trajectory, performed in the MPC-NO-L${}_{1}$ algorithm without any linearisation (Figure 2). Of course, increasing the value of the coefficient λ would solve the problem, but it leads to slower control. The best results are obtained in the MPC-NPLT3-L${}_{1}$ algorithm, in which the trajectory used for linearisation is constant, and all its elements are equal to the value of the process input corresponding to the current output set-point. Such an input value is calculated using an inverse neural static model of the process $u^{\mathrm{sp}}\left(k\right)=\tilde{g}\left(y^{\mathrm{sp}}\left(k\right)\right)$.

**Figure 4:**
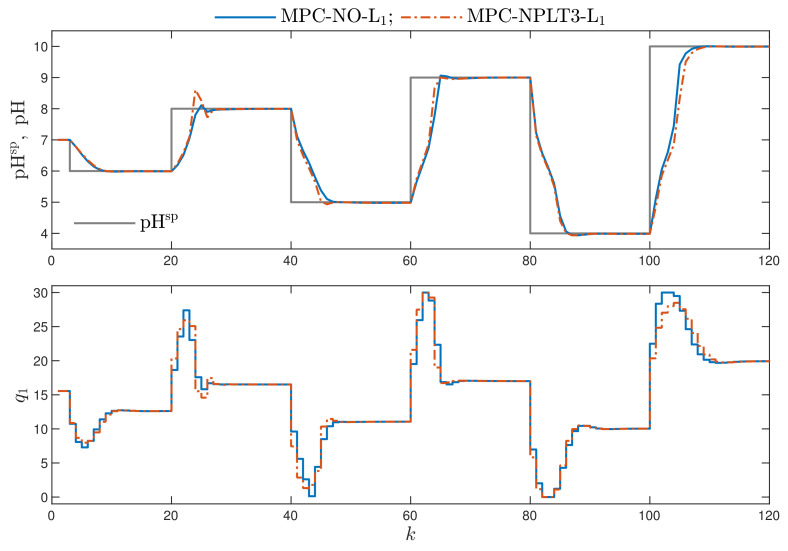
Simulation results: the MPC-NO-L${}_{1}$ and MPC-NPLT3-L${}_{1}$ algorithms.

Let us remind ourselves that our objective is to obtain an MPC algorithm that uses the L${}_{1}$ cost function but it should be computationally efficient, i.e., quadratic optimisation should be used in place of nonlinear optimisation. On the other hand, the “ideal” MPC-NO-L${}_{1}$ algorithm is treated as the reference. In different words, we develop a method that is computationally much simpler, but we hope to obtain control accuracy similar to that possible in the reference MPC-NO-L${}_{1}$ algorithm. Figure 4 compares the best computationally efficient MPC algorithms with one repetition of trajectory linearisation and quadratic optimisation at each sampling instant, i.e., the MPC-NPLT3-L${}_{1}$ scheme, with the reference MPC-NO-L${}_{1}$ scheme. We can see that the MPC-NPLT3-L${}_{1}$ algorithm discussed in this work gives very good trajectories, very similar to those obtained in the MPC-NO-L${}_{1}$ control approach. However, there are some small discrepancies between the obtained trajectories.

**Figure 5:**
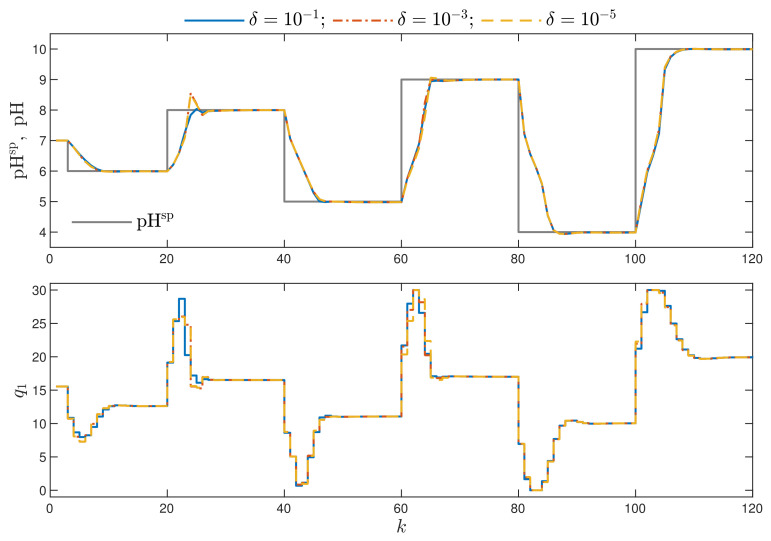
Simulation results: the MPC-NPLPT-L${}_{1}$ algorithm for three values of the parameter δ.

**Figure 6:**
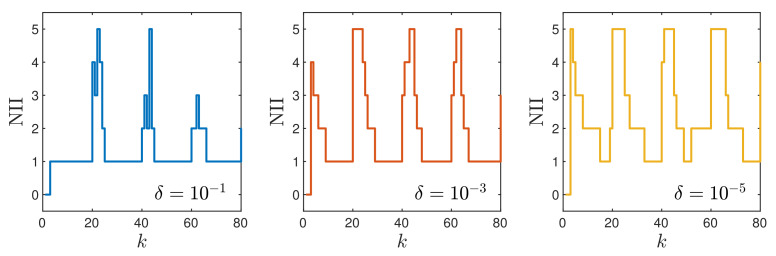
Simulation results: the number of internal iterations (NII) in the consecutive sampling instants of the MPC-NPLPT-L${}_{1}$ algorithm for three values of the parameter δ.

Now, let us consider the MPC-NPLPT-L${}_{1}$ algorithm, in which a few repetitions of trajectory linearisation and quadratic optimisation are possible at each sampling instant. The maximal allowed number of such repetitions is five. The stopping criteria (continuation and termination of the internal iterations) are defined by an additional parameter δ [29]. The internal iterations are continued if the sum of squared differences between the prediction of the model output and the required set-point is greater than δ. The internal iterations are terminated if the sum of squared differences between the optimal solution obtained in two consecutive internal iterations is lower than δ. In general, the lower the parameter δ, the better control quality we expect to obtain, but the number of repetitions of the internal iterations increases. Figure 5 shows the obtained trajectories of the MPC-NPLPT-L${}_{1}$ algorithm for three values of the parameter δ. For the considered neutralisation process, the obtained differences are small, but the best results are obtained for $\delta =10^{-1}$, as it gives the best trajectory for the second set-point step. The considered neutralisation process is nonlinear. The process gain and its dynamics heavily depend on the current operating points. Hence, for different operating points, different overshoot and settling times are observed. Figure 6 depicts the number of internal iterations in the consecutive sampling instants of the MPC-NPLPT-L${}_{1}$ algorithm for three values of the parameter δ. More than one internal iteration is necessary when the set-point changes; when the process is close to the required operating point, one internal iteration is sufficient. It is clear that the lower the parameter δ, the more internal iterations are necessary. This is true provided that a perfect model is used in MPC. In reality, the model is an approximation of the process. In our case, the unavoidable process–model mismatch results in the lowest overshoot for $\delta =10^{-1}$.

**Figure 7:**
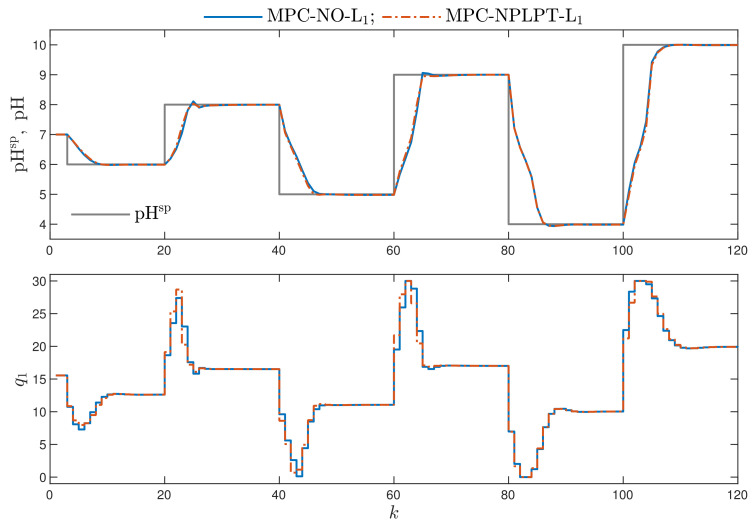
Simulation results: the MPC-NO-L${}_{1}$ algorithm vs. MPC-NPLPT-L${}_{1}$ algorithm.

Remembering our objective the most important issue is to compare the trajectories of the reference MPC-NO-L${}_{1}$ algorithm and the computationally efficient MPC-NPLPT-L${}_{1}$ scheme in the latter one, $\delta =10^{-1}$. Figure 7 shows the obtained trajectories. It is very important to note that the recommended MPC-NPLPT-L${}_{1}$ algorithm with on-line trajectory linearisation and quadratic optimisation gives very similar, practically the same, trajectories as the algorithm with nonlinear optimisation. It is necessary to recall Figure 4, which compares the performance of the MPC-NPLT3-L${}_{1}$ algorithm in which linearisation and optimisation are performed only once at each sampling instant. Comparing Figure 4 and Figure 7, it is clear that multiple linearisation and optimisation possible at each sampling instant in the MPC-NPLPT-L${}_{1}$ approach is really beneficial for the considered process.

**Figure 8:**
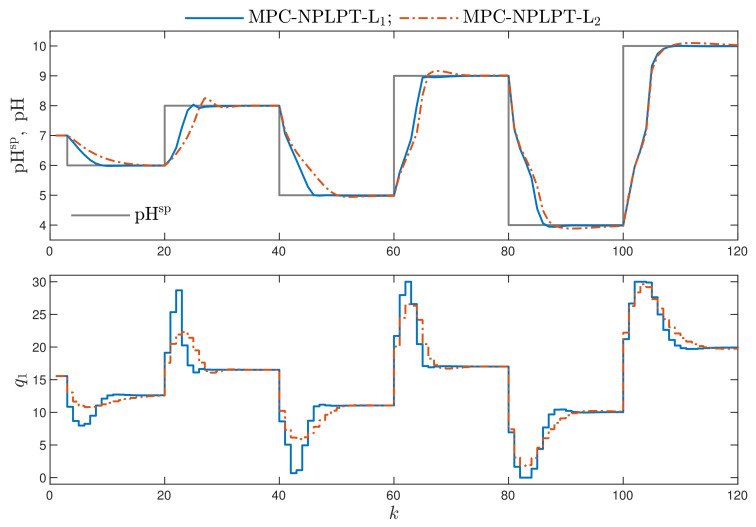
Simulation results: the MPC-NPLPT-L${}_{1}$ algorithm vs. MPC-NPLPT-L${}_{2}$ algorithm.

In Figure 8, the recommended MPC-NPLPT-L${}_{1}$ algorithm, which uses the L${}_{1}$ norm, is compared with its counterpart, MPC-NPLPT-L${}_{2}$, in which the classical L${}_{2}$ norm is used. The use of the L${}_{1}$ norm leads to a lower overshoot and shorter setting time.

Having visually compared the obtained trajectories of the discussed MPC algorithms, it is interesting to analyse their performance and differences using some numerical control quality indicators [70]. We consider the following indices:

- The sum of absolute values of control errors for the whole simulation horizon defined as:
  (50) $$E_{1}=\sum_{k=0}^{120}\left|y^{\mathrm{sp}}\left(k\right)-y\left(k\right)\right|$$
  where y(k) denotes the real value of the process output obtained in simulation.
- The sum of absolute values of differences between the output of the process when it is controlled by the “ideal” MPC-NO-L${}_{1}$ algorithm ($y^{\mathrm{MPC}\text{-}\mathrm{NO}\text{-}L_{1}}\left(k\right)$) and the output of the process when it is controlled by a compared MPC scheme (y(k)). These differences are considered for the whole simulation horizon:
  (51) $$E_{1}^{\mathrm{MPC}\text{-}\mathrm{NO}\text{-}L_{1}}=\sum_{k=0}^{120}\left|y^{\mathrm{MPC}\text{-}\mathrm{NO}\text{-}L_{1}}\left(k\right)-y\left(k\right)\right|$$
- The sum of squared control errors for the whole simulation horizon defined as:
  (52) $$E_{2}=\sum_{k=0}^{120}{\left(y^{\mathrm{sp}}\left(k\right)-y\left(k\right)\right)}^{2}$$
- The sum of squared differences between the output of the process when it is controlled by the “ideal” MPC-NO-L${}_{1}$ algorithm and the output of the process when it is controlled by a compared MPC scheme. These differences are considered for the whole simulation horizon:
  (53) $$E_{2}^{\mathrm{MPC}\text{-}\mathrm{NO}\text{-}L_{1}}=\sum_{k=0}^{120}{\left(y^{\mathrm{MPC}\text{-}\mathrm{NO}\text{-}L_{1}}\left(k\right)-y\left(k\right)\right)}^{2}$$

It is important to stress the fact that the performance criterion $E_{1}$ is not directly minimised in MPC algorithms that use the L${}_{1}$ cost function $J_{1}$ (Equation (4)). This is because the actually minimised cost function $J_{1}$ takes into account the absolute value of control errors predicted over the prediction horizon. The horizon is shifted at each sampling instant. Moreover, it takes into account the second part of the cost function in which the sum of squared moves of the manipulated variable of the control horizon is penalised. Similarly, the performance criterion $E_{2}$ is not directly minimised in MPC algorithms that use the L${}_{2}$ cost function $J_{2}$ (Equation (3)).

Let us state our expectations and objectives. When the norm L${}_{1}$ is used in MPC, we expect to obtain the lowest value of the indices $E_{1}$ and $E_{1}^{\mathrm{MPC}\text{-}\mathrm{NO}\text{-}L_{1}}$. The more similar the obtained trajectories are to those obtained in the MPC-NO-L${}_{1}$ scheme, the closer the index $E_{1}^{\mathrm{MPC}\text{-}\mathrm{NO}\text{-}L_{1}}$ is to 0.

Table 1 presents numerical values of the control quality indices (50)–(53). The following algorithms are considered: MPC-NPLT1, MPC-NPLT2 and MPC-NPLT3 algorithms, three versions of the MPC-NPLPT scheme with different stopping criterion defined by the parameter δ and the MPC-NO approach. The results are divided into two parts: in the first one, the norm L${}_{1}$ is used in MPC; in the second one, the norm L${}_{2}$ is considered; the cost function type minimised in MPC is denoted in colour. In addition to the control quality indices, Table 1 also specifies the relative calculation time of all algorithms; the results are given in percentages in such a way that the calculation time for the most demanding algorithm, i.e., MPC-NO-L${}_{1}$, is treated as 100%. Considering the obtained numerical results, we are able to formulate the following observations concerned with control quality:

1. Comparing the MPC algorithms with the norm L${}_{1}$, in which one trajectory linearisation and quadratic optimisation are executed at each sampling instant, the best results are obtained in the MPC-NPLT3-L${}_{1}$ scheme, in which the trajectory linearisation is performed using an inverse static model of the process. That algorithm gives the lowest values of the performance indices $E_{1}$ and $E_{1}^{\mathrm{MPC}\text{-}\mathrm{NO}\text{-}L_{1}}$. It confirms the comparison given in Figure 3.
2. Better results are possible when a few repetitions of trajectory linearisation and quadratic optimisation are possible at each sampling instant in the MPC-NPLPT-L${}_{1}$ scheme. The obtained value of the indices $E_{1}$ and $E_{1}^{\mathrm{MPC}\text{-}\mathrm{NO}\text{-}L_{1}}$ are lower. It confirms the comparison given in Figure 7. Moreover, the lower the parameter δ, the more similar the obtained trajectory is to that possible in the reference MPC-NO-L${}_{1}$ scheme.
3. Bearing in mind our expectations and objectives, the classical MPC algorithms that use the L${}_{2}$ norm give a worse performance. This confirms the comparison given in Figure 8. For the corresponding algorithms, the values of both $E_{1}$ and $E_{1}^{\mathrm{MPC}\text{-}\mathrm{NO}\text{-}L_{1}}$ performance indices are better (i.e., lower) when the norm L${}_{1}$ is used; the norm L${}_{2}$ gives worse results. This effect is best visible when we consider the $E_{1}^{\mathrm{MPC}\text{-}\mathrm{NO}\text{-}L_{1}}$ performance index. For example, comparing the MPC-NPLPT-L${}_{1}$ and MPC-NPLPT-L${}_{2}$ algorithms with $\delta =10^{-5}$, that index is in the first case approximately 11 times lower.
4. It is very interesting that the use of the L${}_{1}$ norm in place of the classical L${}_{2}$ one leads to not only better (lower) values of the indices $E_{1}$ and $E_{1}^{\mathrm{MPC}\text{-}\mathrm{NO}\text{-}L_{1}}$, which is natural, but also makes it possible to reduce the indices $E_{2}$ and $E_{2}^{\mathrm{MPC}\text{-}\mathrm{NO}\text{-}L_{1}}$. For all pairs of algorithms (with L${}_{1}$ and L${}_{2}$ norms), the $E_{2}$ index is slightly lower when the L${}_{1}$ norm is used. This difference is even more clear when we consider the $E_{2}^{\mathrm{MPC}\text{-}\mathrm{NO}\text{-}L_{1}}$ index. It confirms the comparison given in Figure 8.

Furthermore, it is also possible to put forward the following observations concerned with computation time:

1. In general, all MPC algorithms with the norm L${}_{1}$ are more computationally demanding than their counterparts that use the norm L${}_{2}$. This is because, in the first case, in all calculations, i.e., in prediction, linearisation and optimisation, the neural approximator determines the absolute values of the control errors over the prediction horizon whereas, in the second case, no approximator is used, the predictions and control errors are used directly in all calculations.
2. All MPC algorithms with linearisation and quadratic optimisation are less computationally demanding than the reference “ideal” MPC-NO algorithm.
3. The more complicated the trajectory linearisation, the longer the calculation time. The lowest calculation time is observed in the MPC-NPLT1, MPC-NPLT2 and MPC-NPLT3 algorithms, with one repetition of linearisation and quadratic optimisation at each sampling instant. The calculation time becomes longer in the MPC-NPLPT scheme, with a few repetitions of linearisation and optimisation at each instant; the lower the parameter δ, the longer the calculation time.

**Figure 9:**
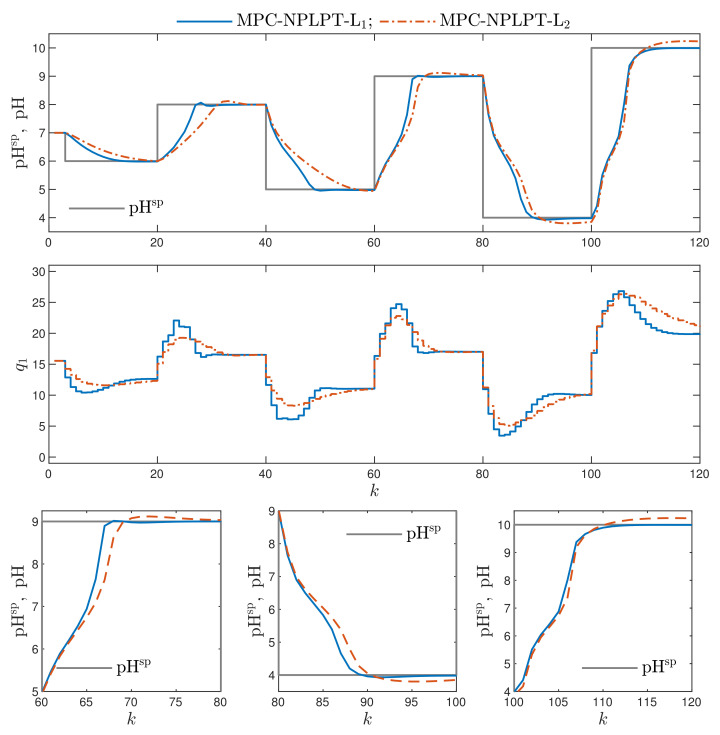
Simulation results: the MPC-NPLPT-L${}_{1}$ algorithm vs. MPC-NPLPT-L${}_{2}$ algorithm, in both cases λ=5; two top panels show the results for the whole simulation horizon, three bottom panels show enlarged fragments for the sampling instants 60≤k≤80, 80≤k≤100 and 100≤k≤120.

Finally, let us compare the results of the MPC-NPLPT-L${}_{1}$ and MPC-NPLPT-L${}_{2}$ algorithms when the penalty coefficient is increased. In our study, the value λ=5 is considered. Figure 9 presents the obtained trajectories. Two top panels show the results for the whole simulation scenario, whereas the bottom ones show enlarged fragments for three set-point changes. It is interesting to see an additional advantage of the recommended L${}_{1}$ norm because, in such a case, the overshoot is much lower and the setting time is much shorter when compared with the trajectories possible when the classical L${}_{2}$ norm is used. In the considered comparison, for λ=5, the benefits of using the L${}_{1}$ norm are even more clear than in the case of the default value of the penalty coefficient, i.e., λ=1, as presented in Figure 8.

## 5. Conclusions

The presented MPC-L${}_{1}$ algorithms have three essential advantages. Firstly, thanks to using a neural differentiable approximator of the non-differentiable absolute value function and on-line advanced trajectory linearisation, computationally simple quadratic optimisation is used in place of demanding nonlinear optimisation. Secondly, the obtained trajectories are very similar, practically the same, as those possible in the reference scheme with nonlinear optimisation. Thirdly, it is shown that the use of the recommended L${}_{1}$ norm gives better results defined using different control quality criteria, such as the sum of absolute values or squared control errors, overshoot and setting time. Furthermore, it must be stressed that the L${}_{1}$ norm even gives better results than the classical L${}_{2}$ one in terms of the classical control performance indicator that measures squared control errors. The discussed MPC is very universal, it may be used in industrial process control applications and in fast embedded systems. The only condition is that the model used for prediction and the neural approximator must be differentiable.

As future works, the following issues are worth investigating: taking into account not only constraints imposed on the manipulated variable, but also on the predicted controlled one, the development of nonlinear MPC-L${}_{1}$ algorithms for multivariable processes with multiple inputs and multiple outputs, considering state–space process description in place of the input–output one and considering alternative types of the cost function, not only the discussed L${}_{1}$ norm. 

## Figures and Tables

**Table 1:** Simulation results: the values of the control quality criteria and the scaled calculation time, the cost function type corresponding to that minimised in MPC is denoted in color; in all cases λ=1.

Algorithm	$E_{1}$	$E_{1}^{\mathrm{MPC}\text{-}\mathrm{NO}\text{-}L_{1}}$	$E_{2}$	$E_{2}^{\mathrm{MPC}\text{-}\mathrm{NO}\text{-}L_{1}}$	Calculation Time
MPC-NPLT1-L${}_{1}$	7.8818 × 10^1^	1.0431 × 10^1^	2.2752 × 10^2^	4.4144	34.00%
MPC-NPLT2-L${}_{1}$	7.8897 × 10^1^	1.5057 × 10^1^	2.1985 × 10^2^	9.3569	33.7%
MPC-NPLT3-L${}_{1}$	7.1167 × 10^1^	7.4706	2.2197 × 10^2^	3.6258	33.6%
MPC-NPLPT-L${}_{1}$, $\delta =10^{-1}$	6.9590 × 10^1^	2.7467	2.1626 × 10^2^	3.3734 × 10^−1^	40.3%
MPC-NPLPT-L${}_{1}$, $\delta =10^{-3}$	6.9768 × 10^1^	2.6469	2.1435 × 10^2^	8.7502 × 10^−1^	49.3%
MPC-NPLPT-L${}_{1}$, $\delta =10^{-5}$	7.0350 × 10^1^	1.5524	2.1573 × 10^2^	5.4643 × 10^−1^	60.8%
MPC-NO-L${}_{1}$	7.0371 × 10^1^	–	2.1631 × 10^2^	–	100.0%
MPC-NPLT1-L${}_{2}$	8.6977 × 10^1^	1.8184 × 10^1^	2.3746 × 10^2^	8.9869	21.0%
MPC-NPLT2-L${}_{2}$	8.3845 × 10^1^	1.6043 × 10^1^	2.2758 × 10^2^	5.8777	20.8%
MPC-NPLT3-L${}_{2}$	8.3647 × 10^1^	1.5071 × 10^1^	2.3459 × 10^2^	6.2455	21.0%
MPC-NPLPT-L${}_{2}$, $\delta =10^{-1}$	8.3784 × 10^1^	1.4916 × 10^1^	2.3559 × 10^2^	5.5591	23.6%
MPC-NPLPT-L${}_{2}$, $\delta =10^{-3}$	8.4162 × 10^1^	1.6693 × 10^1^	2.2885 × 10^2^	6.6640	30.9%
MPC-NPLPT-L${}_{2}$, $\delta =10^{-5}$	8.4795 × 10^1^	1.7317 × 10^1^	2.2976 × 10^2^	7.2166	35.6%
MPC-NO-L${}_{2}$	8.5089 × 10^1^	1.7708 × 10^1^	2.3033 × 10^2^	7.6448	73.3%

## Data Availability

Not applicable.
